# Frequency and genotype of human papillomavirus among Sudanese patients with head and neck tumours

**DOI:** 10.3332/ecancer.2012.282

**Published:** 2012-12-04

**Authors:** Hussain Gadelkarim Ahmed, Saadalnour Abusail Mustafa, Faris Margani Eltom, Ali Yousef Yahia Babiker

**Affiliations:** 1 Department of Pathology, College of Medicine, University of Hail, Hail, Kingdom of Saudi Arabia; 2 Department of Histopathology and Cytology, Faculty of Medical Laboratory Sciences, Elneelain University, Khartoum, Sudan; 3 College of Applied Medical Science, Tyba University, Kingdom of Saudi Arabia; 4 College of Applied Medical Science, Aqasseem University, Al-Qassim, Kingdom of Saudi Arabia

**Keywords:** head and neck cancer, human papillomavirus, Sudanese

## Abstract

**Objectives:**

The aim of this study was to determine the frequency and genotype of human papillomavirus (HPV) infections in head and neck squamous cell carcinomas (HNSCCs) and benign head and neck tumours.

**Methodology:**

A retrospective study was performed on 150 samples of patients diagnosed with HNSCCs and 50 samples obtained from patients diagnosed with benign head and neck tumours. Tumour DNA was amplified using polymerase chain reaction (PCR) with HPV consensus and multiplex primers.

**Results:**

Six of the 150 (4%) HNSCCs were HPV positive. HPV16 was the most prevalent type, with single infections present in 3/6 (50%) cases, whereas HPV18 and HPV33 were detected in 2/6 (33%) and 1/6 (17%), respectively. HPV infections were detected in 3 (50%) cases of oral cavity and 3 (50%) cases of pharynx.

**Conclusions:**

There was a significant association between HPV infection and HNSCCs (*P* < 0.05). The present data support the importance of HPV infection in oral and larynx tumours.

## Introduction

Head and neck squamous cell carcinoma (HNSCC) is the seventh most commonly diagnosed cancer worldwide [1] and is associated with survival rates. Its incidence varies widely among different regions. In North America and Europe, HNC accounts for 3–4% of all cancer diagnoses. Conversely, in Southeast Asia and Africa, HNC accounts for approximately 8–10% of all cancers [2]. HNC has traditionally been linked to alcohol and tobacco abuse [3].

However, 15–20% of HNC cases have no known tobacco or alcohol exposure [4, 5], thus, other agents, such as viruses, are being investigated. It is now evident that a significant proportion of HNSCCs are caused by HPV [6]. High-risk HPV subtype 16 accounts for more than 85% of all HPV-positive (HPV+) tumours in HNSCC [7]. Patients with HPV-positive HNSCC had a lower risk of dying and a lower risk of recurrence than HPV-negative HNSCC patients [8].

Knowledge of HPV and EGFR status can have implications for treatment options and prognosis in HNSCC. Therefore, the aim of this study was to determine the frequency and genotype of human HPV infections in HNSCCs and benign head and neck tumours amongst Sudanese patients using polymerase chain reaction (PCR).

Polymerase chain reaction is a selective target amplification assay capable of exponential and reproducible increase in the PV sequences present in biological specimens. The amplification process can theoretically produce one billion copies from a single double stranded DNA molecule after 30 cycles of amplification. The sensitivity and specificity of PCR-based methods can vary, depending on the: DNA extraction procedures; site and type of clinical sample; sample transport and storage; primer sets; the size of the PCR product; reaction conditions and performance of the DNA polymerase used in the reaction; the spectrum of HPV DNA amplified; and ability to detect multiple types. Generally, a sensitivity of 1–10 copies per PCR reaction is achieved by most methods utilized [9].

## Materials and methods

A total of 200 patients, 125 males and 75 females (male/female ratio, 1.7:1) aged between 9 and 85 years with a mean age of 51 years, were diagnosed as having HNSCCs (150 patients) or benign head and neck lesions (50 patients). They were investigated for the presence of different HPV genotypes. The diagnosis was based on clinical examination and histological features of the biopsy. HNSCCs were graded as well, moderate, and poorly differentiated using Royal College of Pathologists criteria [10]. Benign lesions include: 17 squamous papilloma, 13 pyogenic granuloma, 7 reactive hyperplasia, 5 dysplasia, 5 inflammations, and 3 pleomorphic adenoma.

The sample included full coverage of patients with HN lesions referred to our hospital within a two-year timeframe. Ethical consent was obtained from the ethical committee of the Faculty Research Board and Hospital.

## DNA extraction

Genomic DNA was isolated from the formalin-fixed paraffin embedded tissue (FFPET) specimens for which adjacent sections were examined by microscopy to assess the presence of adequate tumour tissue and the proportion of stromal tissue. All HN lesion samples used for DNA extraction showed >60% tumour tissue in each case. Cellular DNA was extracted from each paraffin-embedded tissue block using 30–50 μ sections. DNA was extracted by DNA extraction kit purchased from *Sacace biotechnologies-Casera—Italy*. The standard protocol of the kit was performed with the following modifications [11]. First, the step of xylene/ethanol extraction of the wax was eliminated. Second, the lysis buffer of each kit was added directly to the tissue section in a microcentrifuge tube. Third, the tissue section immersed in lysis buffer was heated to 98°C for 15 min before the addition of proteinase K. Briefly, in the HighPure DNA preparation kit, 200 µl of lysis buffer was added to a tissue sample in a 1.5-ml microcentrifuge tube. The microcentrifuge tube was then placed in a heating block at 98°C for 15 min and was briefly cooled at room temperature for 5 min. Proteinase K solution (40 μl of 20 mg/ml) was added to the heat-treated tissue section. The tissue sample was incubated at 68°C for 45 min. Binding buffer (200 μl) was added to the sample, and the sample was incubated for 10 min at 72°C. After incubation, 100 μl isopropanol was added to the sample. The sample mixture was transferred to a HighPure Tube Assembly. The genomic DNA was retained on the column and washed twice with 500 μl of wash buffer before eluting with 200 μl of 10 mmol/l Tris–HCl (pH 8.0). For the DNA extraction kit, 180 μl of buffer ATL was added to a tissue sample in a 1.5-ml microcentrifuge tube. The microcentrifuge tube was then placed in a heating block set at 98°C for 15 min and briefly cooled at room temperature for 5 min. Proteinase K solution (20 μl) was added to the heat-treated tissue section. The tissue sample was incubated at 68°C for 45 min. Buffer AL (200 μl) was added to the sample, followed by incubation for 10 min at 72°C. After incubation, 200 μl ethanol was added to the sample, and the mixture was transferred to Spin Column. The genomic DNA was retained on the column and washed twice with 500 μl of wash buffer before eluting with 200 μl of 10 mmol/l Tris–HCl (pH 8.0) at room temperature.

## Polymerase chain reaction (PCR)

Total cellular DNA (100 ng/μl) was amplified by PCR. HPV types (16, 18, 31, 33, 35, 39, 45, 51, 52, 56, 58, 59) specific primers were used for conventional mutiplex PCR as indicated in Table 1. These primers were designed to detect E7 and E6 open reading frame of HPV. One microlitre (100 ng/μl) of DNA was mixed with 50 μl PCR mix [125 mM dNTPs and 0.5 units of Red Hot Taq polymerase, 50 mM KCl, 10 mM Tris–HCl pH 8.3, 2 mM MgCl2 and 200 mg/ml bovine serum albumin (BSA)]. The PCR was initiated by hot start at 95°C for 5 min, then 30 cycles (denaturation 95°C/40 s; annealing 50°C/60 s; and extension 72°C/90 s). Then one last step for extension at 72°C for 10 min (Table 2). Ten microlitres of the PCR product was mixed with two loading solutions in 2% agarose gel electrophoresis and run for 60 min, then stained by ethidium bromide and photographed by a gel documentation system (Gel mega, digital camera and software in a computer).

The identification of each HPV type present was achieved by determining the size of the PCR amplification product by gel electrophoresis.

## Results

We tested 200 HNSCC tumour samples (150 HNSCCs and 50 benign tumours) for the presence of HPV DNA with PCR. HPV genomic material using E6 and E7 primers were detected in 6/150 (4%) of HNSCCs [3/6 (50%) HPV-16, 2/6 (33%) HPV-18, and 1/6 (17%) HPV-33]. All of the 50 benign samples were found negative for HPV. Consequently, the risk associated with HPV infection was found to be statistically significant (*P* < 0.05 and relative risk = 1.2531). Four (66.7%) HPV positive were found among males and the remaining two (43.3%) were found among females. Two HPV-16, one HPV-18, and one HPV-33 were detected among males, whereas one HPV-16 and one HPV-18 were detected among females.

**Figure 1:**
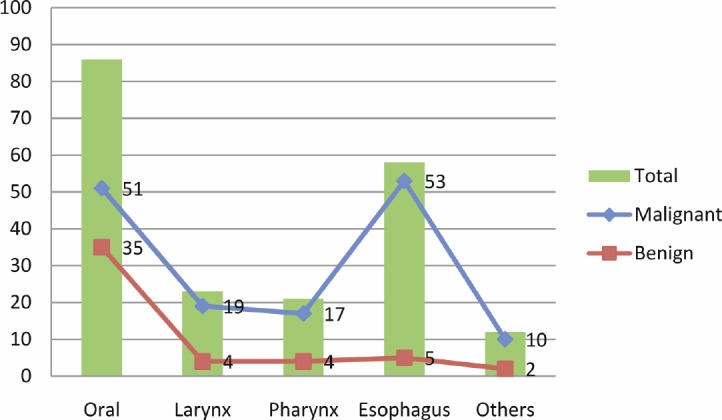
Description of study subjects by the site of HN tumour.

The description of the tumour site is described in Figure 1. The majority of HN tumours originated from the oral cavity and oesophagus, particularly HNCCs; hence, most of the benign tumours were seen in the oral cavity. Notably, all of the HPV positive samples were found in oral cavity (33.3%) and in the larynx (66.7%). Of the three HPV-16, one was detected in the oral cavity and the remaining two were detected in the larynx. Of the two positive cases of HPV-18, one was detected in the oral cavity and the other in the larynx. The only one case of HPV-33 was detected in the larynx.

**Figure 2:**
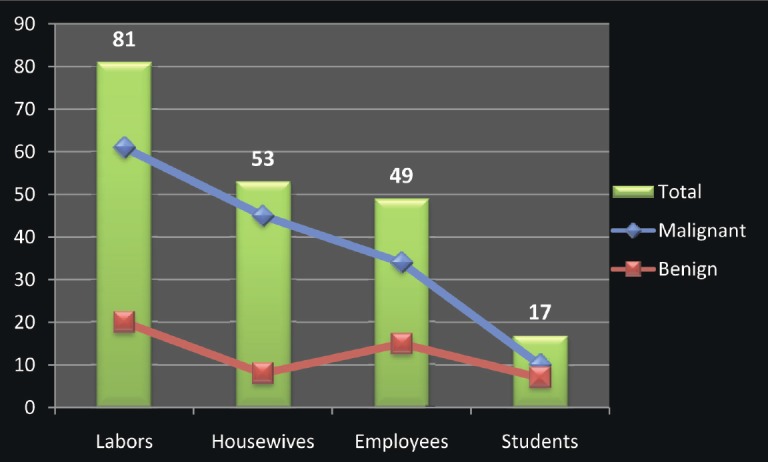
Description of the study population by occupation.

Regarding occupation, most of the patients were labourers followed by housewives, employees, and students, as indicated in Figure 2. Positive-HPV samples were identified among 2/81 (2.5%), 2/53 (3.8%), and 2/49 (4.1%) of the labourers, housewives, and employees, respectively. The three positive HPV-16 were identified among labourers, housewives, and employees; the two cases of HPV-18 were demonstrated among housewives and employees; and the case of HPV-33 was identified among employees.

**Figure 3:**
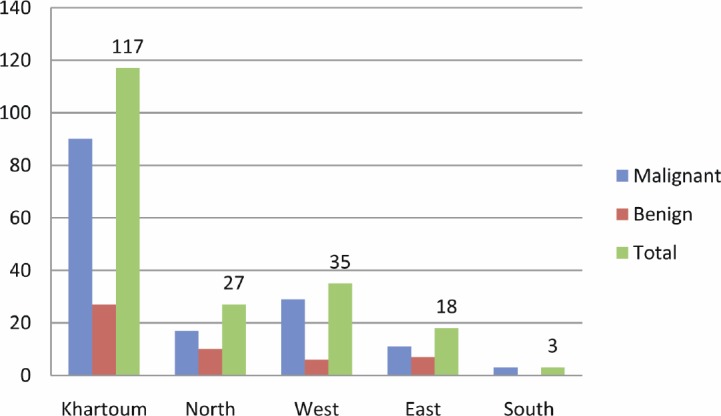
Description of the study population by residence.

The majority of patients were from Khartoum followed by the western regions, as shown in Figure 3. Two of the HPV-16, one of the HPV-18, and one of the HPV-33 positive cases were identified among patients from Khartoum. The remaining HPV-16 was found in East and HPV-18 was found in the West.

**Figure 4:**
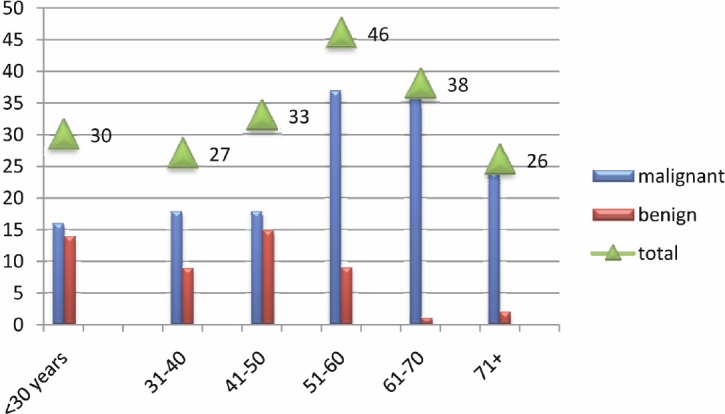
Description of the study population by age.

HNCCS were increasingly seen amongst the older population, particularly among age groups of 51–60 and 61–70 years as indicated in Figure 4. However, HPV positive cases were predominantly seen in the younger age group of 31–40 years representing four (66.7%). The remaining two (43.3%) were found among the age group of 70+.

## Discussion

HPV infection is emerging as an important risk factor for several HNSCCs. Previous studies have shown that patients with HPV positive tumours actually benefit from a better overall disease-specific survival than patients with HPV-negative tumours [12]. In this study, we used E6 and E7 as the two key viral oncoproteins that induce and propagate cellular transformation [13]. The current study has indicated a significant association between HPV infection and HNSCCs in Sudan, and to the best of our knowledge this is the first report in this context from Sudan. In a study investigating the prevalence of HPV, in 155 OSCCs from eight different countries from different ethnic groups, continents, and with different socioeconomic backgrounds, the highest prevalence of HPV was seen in Sudan (65%) [14].

However, there are some studies investigating the relationship between oral cancer and HPV infection. Of these studies, a study found that HPV was in only two Sudanese cases, both of which harboured types 6 and 11: these two cases demonstrated mild epithelial dysplasia [15]. Another study evaluated the possible role of high risk HPV 16 and 18 in oral squamous cell carcinomas (OSCC), 40 SCCs, and 15 benign lesions, HPVDNA was detected in 15% of cases (6 of 40 cases) and none of controls (*n* = 15), *P* < 0.0001 [16].

In the present study, all of the positive samples were identified in oral and larynx sites, and most types identified were HPV16 and HPV18, particularly in the oral tissues. HPV infections are commonly identified in the tumour tissues of patients with head and neck SCCs, in which HPV16 and 18 are the most prevalent HPV genotypes [17]. Although, the study from Sudan [16] showed that HPV18 is more prevalent in the OSCCs than HPV16, but many studies from other countries have revealed the domination of HPV16 in HNSCCs in general and OSCCs in particular [18–21].

According to occupation, most HPV infections were found among labourers followed by housewives. However, a link has been demonstrated between social class and HPV-related cancers. Data indicate that cervical cancer incidence is considerably higher among women of working age in manual than in non-manual classes [22, 23]. The impact of occupational exposures, together with the occupational circumstances and industrial areas where exposures to carcinogenic agents occurred in the past, on population cancer morbidity and mortality can be compared with the impact of other causes of cancer [22].

As the results show, most patients with positive HPV were from Khartoum. This might be attributed to the fact that Khartoum harbours a diverse population from all over Sudan, with varying socioeconomic status and behavioural differences.

Most HPV positive cases in the present study were aged 31–40, and men accounted for over 74%. Oral cancer in Sudan is lower among females [24]. This is because toombak use (synergistic factor to HPV) is uncommon among females, as it is considered as a social stigma in the Sudan. However, HPV-associated oropharyngeal cancers generally are diagnosed at slightly younger ages in men than in women [25].

One of the limitations of the study is that we only determined the presence of HPV DNA in human specimens, which is not conclusive proof for an active viral infection. Another limitation is that we applied a single template molecule, which is less specific compared to nested PCR in which two pairs of PCR primers were used for a single locus. The first pair amplified the locus as seen in any PCR experiment. The second pair of primers (nested primers) binds within the first PCR product and produces a second PCR product that will be shorter than the first one. The logic behind this strategy is that if the wrong locus were amplified by mistake, the probability is very low that it would also be amplified a second time by a second pair of primers.

The current study provides support for the contributing role of HPV (particularly types 16, 18, 33) infection in the etiology of HNSCCs in Sudan.

## Figures and Tables

**Table 1.** Sequences of type-specific PCR primers used in this study.

HPV genotype	Sequence (5′–3′)	Amplification (bp)
16	CAC AGT TAT GCA CAG AGC TGC	457
18	CAC TTC ACT GCA AGA CAT AGA	322
31	GAA ATT GCA TGA ACT AAG CTC G	263
33	ACT ATA CAC AAC ATT GAA CTA	398
35	CAA CGA GGT AGA AGA AAG CAT C	358
39	GAC GAC CAC TAC AGC AAA CC	280
45	GTG GAA AAG TGC ATT ACA GG	151
51	GAG TAT AGA CGT TAT AGC AGG	223
52	TAA GGC TGC AGT GTG TGC AG	229
56	GTG TGC AGA GTA TGT TTA TTG	181
58	GTA AAG TGT GCT TAC GAT TGC	274
59	CAA AGG GGA ACT GCA AGA AAG	215

**Table 2.** Show PCR Program used for amplification of HPV genes

steps	Temperature	time	Cycles
0	95°C	Pause	
1	95°C	15 min	1
2	95°C	15 sec	42
	65°C	40 sec	
	72°C	20 sec	
3	72°C	1 min	1
4	4°C	storage	
